# Multi-objective optimization of tumor response to drug release from vasculature-bound nanoparticles

**DOI:** 10.1038/s41598-020-65162-2

**Published:** 2020-05-19

**Authors:** Ibrahim M. Chamseddine, Hermann B. Frieboes, Michael Kokkolaras

**Affiliations:** 1 Deparment of Integrated Mathematical Oncology, Moffitt Cancer Center & Research Institute, Tampa, FL USA; 2 Department of Bioengineering, University of Louisville, Louisville, KY USA; 3 James Graham Brown Cancer Center, University of Louisville, Louisville, KY USA; 4 Center for Predictive Medicine, University of Louisville, Louisville, KY USA; 5 Department of Mechanical Engineering, McGill University, Montreal Quebec, Canada; 6 GERAD – Group for Research in Decision Analysis, Montreal Quebec, Canada

**Keywords:** Biomedical engineering, Cancer therapy

## Abstract

The pharmacokinetics of nanoparticle-borne drugs targeting tumors depends critically on nanoparticle design. Empirical approaches to evaluate such designs in order to maximize treatment efficacy are time- and cost-intensive. We have recently proposed the use of computational modeling of nanoparticle-mediated drug delivery targeting tumor vasculature coupled with numerical optimization to pursue optimal nanoparticle targeting and tumor uptake. Here, we build upon these studies to evaluate the effect of tumor size on optimal nanoparticle design by considering a cohort of heterogeneously-sized tumor lesions, as would be clinically expected. The results indicate that smaller nanoparticles yield higher tumor targeting and lesion regression for larger-sized tumors. We then augment the nanoparticle design optimization problem by considering drug diffusivity, which yields a two-fold tumor size decrease compared to optimizing nanoparticles without this consideration. We quantify the tradeoff between tumor targeting and size decrease using bi-objective optimization, and generate five Pareto-optimal nanoparticle designs. The results provide a spectrum of treatment outcomes – considering tumor targeting vs. antitumor effect – with the goal to enable therapy customization based on clinical need. This approach could be extended to other nanoparticle-based cancer therapies, and support the development of personalized nanomedicine in the longer term.

## Introduction

Chemotherapy is the treatment of choice to control metastatic cancer - a stage often reported in patients at the time of clinical presentation. Unfortunately, patients undergoing chemotherapy may have low median survival, especially for pancreatic, lung, and liver cancer^1^. Negative response to treatment is attributed to a number of factors, including tumor microenvironmental barriers, evolution of resistance to drug, and chemotherapeutic toxicity. It has been shown that nanoparticle-mediated drug delivery may substantially enhance the pharmacokinetics of anticancer drugs while addressing some of these factors^2^. However, while many nano-based formulations have undergone pre-clinical and clinical evaluation, few have been translated to the clinic^3^.

The targeting potential of nanotherapy is strongly associated with nanoparticle biophysical and biochemical properties^4–6^. These properties include size^7^, shape (e.g., sphere or ellipsoid)^5^, stiffness^4,8^, and binding affinity of nanoparticle surface ligands to receptors upregulated in cells in tumors^5^. Computational studies have investigated the effect of these properties on treatment efficacy in an attempt to find nanoparticles optimized for maximal anti-tumor activity. Nanoparticle margination^6^ and adhesion to tumor vasculature^5^ have been modeled as a function of nanoparticle properties (size, aspect ratio, ligand surface density, and ligand-receptor binding affinity). Uncertainties in ligand surface density and ligand-receptor affinity have been quantified and incorporated in a nanoparticle-tumor adhesion model using a Bayesian hierarchical approach^9^. Uncertainties in tumor vessel diameter, flow velocity, and hematocrit were considered in a nanoparticle transport model, showing that the transport and dispersion of nanoparticles can be very sensitive to the tumor microvasculature^10^. A continuous-discrete model of nanoparticle-mediated drug delivery in heterogeneously vascularized tumors was presented in^11^. Building upon the spatial tumor model in^12^, the nanoparticle delivery model was previously extended to study the influence of interstitial pressure^13^, hypo-vascularization^14^, tumor acidity^15^, immune activity^16–18^, and vascular density^19,20^ on heterogeneous nanoparticle uptake and tumor response. Further background on mathematical modeling of nanoparticle-mediated drug delivery can be found in^21–24^ and references therein. Of particular relevance to the study here, the transport of tumor vasculature-adhering nanoparticles of differing diameters was modeled in^7^ and the resulting drug response was simulated in^25^.

In a recent study, we utilized rigorous derivative-free optimization to obtain optimal nanoparticle diameters and aspect ratios that maximize nanoparticle accumulation and spatial distribution in tumor tissue using a continuous 2D model of blood flow, nanoparticle accumulation, and drug transport^26^. We quantified the tradeoff between maximizing tumor nanoparticle accumulation and maximizing tumor tissue exposed to drug. The results yielded a set of optimal nanoparticle sizes and aspect ratios. We then integrated the spatial tumor model reported in^25^ and conducted numerical optimization studies to obtain nanoparticle sizes that maximize tumor targeting while minimizing tumor diameter^27^. However, the tradeoff between these two competing objectives was not quantified, and drug potency was treated as a parameter to be controlled in order to relax the competing effects of treatment. In this work, we address the issue of competing objectives and perform optimization studies to increase treatment efficacy beyond what was previously achieved. In particular, we increase the robustness of the nanoparticle design optimization framework by considering drug properties in the nanoparticle design and the tradeoff between treatment efficacy and toxicity.

## Results

### Optimizing nanoparticle design for a cohort of tumors

Clinically, tumor size is an indication of cancer stage, which is primarily used for tumor stratification^28^. Tumors of different sizes are expected to respond differently to treatment. Tumor growth results in changes in vascular density^29^, cell organization^30^, and ECM stiffness^31^, all of which impact drug pharmacodynamics. This has promoted preclinical investigations of the effect of tumor size on treatment selection. Approaches used include grouping tumors by size, utilizing machine learning (e.g.^32,33^,), and designing treatments for average tumors and studying treatment robustness (e.g., with immunotherapy protocols^34^). Here, we seek to optimize treatment based on tumor size at the time of treatment.

The nanoparticle diameter *d* and avidity *α* are used as suitable treatment parameters that can be optimized to maximize treatment efficacy. A cohort of 8 tumors grown to different sizes is considered. Two optimization problems are then formulated and solved: one to minimize ratio of tumor diameter after treatment to diameter at start of treatment (TD) and one to maximize the percent of injected nanoparticles that accumulate in the tumor (TNP).

1 $$\begin{array}{cc}\mathop{min}\limits_{d,\,\alpha }\, & TD\\ {\rm{s}}{\rm{u}}{\rm{b}}{\rm{j}}{\rm{e}}{\rm{c}}{\rm{t}}\,{\rm{t}}{\rm{o}} & \begin{array}{c}1\le d\le 1000\,(nm)\\ {10}^{10}\le \alpha \le {10}^{12}\,({m}^{-2})\end{array}\end{array}$$

2 $$\begin{array}{cc}\mathop{max}\limits_{d,\,\alpha }\, & TNP\\ {\rm{s}}{\rm{u}}{\rm{b}}{\rm{j}}{\rm{e}}{\rm{c}}{\rm{t}}\,{\rm{t}}{\rm{o}} & \begin{array}{c}1\le d\le 1000\,(nm)\\ {10}^{10}\le \alpha \le {10}^{12}\,({m}^{-2})\end{array}\end{array}$$

**Figure 1:**
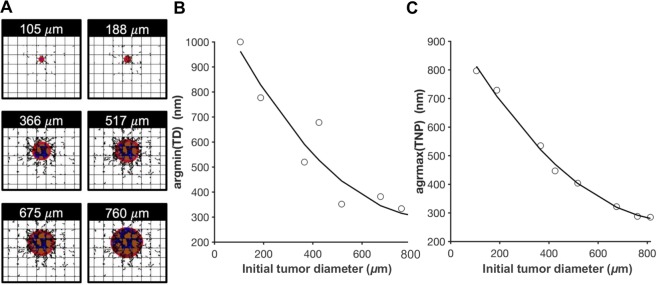
Optimizing nanoparticle designs for tumors of different sizes. (**A**) Tumors of different sizes at which the treatment starts. Change in optimal nanoparticle diameters that (**B**) minimizes tumor size after one day of treatment and (**C**) maximizes percent accumulation of nanoparticles in tumor tissue as a function of tumor size at start of treatment. Black lines represent the second order polynomial fit obtained by minimizing the least linear square. Red: proliferating tumor tissue; blue: hypoxic tissue; brown: necrotic tissue. Orthogonal grid represents pre-existing capillary network surrounding the growing tumor. Irregular lines simulate capillary growth in response to a net balance of pro-angiogenic factors released by hypoxic tumor tissue.

Eq. (1) finds the values of $d$ and $\alpha$ that minimize the tumor diameter ratio. Similarly, Eq. (2) finds the values of the 2 design variables ($d$ and $\alpha$) that maximize the tumoral nanoparticle accumulation. The inequalities in both equations define the upper and lower bounds of each design variable. The two optimization problems in Eqs. (1) and (2) are solved for each tumor in the cohort (Fig. 1(A)). The optimal nanoparticle diameters that minimize tumor size are shown in Fig. 1(B), revealing a monotonically decreasing relation between nanoparticle and tumor diameters; smaller nanoparticles yield more tumor regression than larger particles when the initial tumor size is large. Physically, this means that larger tumors would benefit from targeting by small nanoparticles to allow adequate time for them to circulate through the vasculature and reach the tumor core. The rationale is that the nanoparticle size affects the interaction area between the nanoparticle surface and the blood vessel wall, the number of ligand-receptor pairings, and the hemodynamic force and torque exerted on the nanoparticle. Accordingly, large nanoparticles have generally strong binding affinities to the endothelial layer^5,26,27^, and due to this interaction, they tend to adhere to the vessel walls at the tumor periphery^26^. In contrast, smaller nanoparticles have longer circulation times^35,36^ that allow them to reach the tumor core. A similar pattern was obtained for the maximizers of TNP as shown in Fig. 1(C). A higher percentage of smaller nanoparticles adhere at the vessels of large tumors. This trend could be caused by an increase in vascularization as the tumor progresses. The values of the optimal solutions for both optimization studies are listed in Table 1. Surprisingly, the optimal value of nanoparticle avidity is consistently at the lower bound, in agreement with the results obtained in^27^ and highlighting that low nanoparticle avidity is optimal despite changes in initial tumor size. Although TD and TNP are competing objective functions, as demonstrated in^27^, the optimizers of both objectives follow a decreasing function with respect to tumor size. If the two curves in Fig. 1(B,C) are combined in the same plot, the area encapsulated between them is a tradeoff region. For instance, a tumor with an initial diameter of 760 μm is minimized when targeted with nanoparticles of diameter argmin(TD) = 334 nm. The nanoparticle accumulation administered to such a tumor is maximized when the nanoparticle diameter is argmax(TNP) = 288 nm. Selecting a nanoparticle with diameter between 288 nm and 334 nm presents a tradeoff between TD and TNP. This tradeoff is quantified below.

**Table 1:** Optimal nanoparticle diameters obtained by solving the optimization problems in Eqs. (1) and (2) for the chosen cohort of tumors. Asterisks indicate optimal values. TD: ratio of tumor diameter after treatment to diameter at start of treatment; TNP: percent of injected nanoparticles that accumulate in tumor tissue.

Tumor diameter at start of treatment (μm)	argmin(TD) (nm)	TD* (%)	argmin(TNP) (nm)	TNP* (%)
105	1000	125	797	7.5e-3
188	777	75	729	0.45
366	520	45	535	5.4
425	678	40	447	4.6
517	352	26	404	7.4
675	382	15	322	9.9
760	334	51	288	13.7
813	276	57	285	20.5

### Optimizing nanoparticle design along with drug diffusivity

Nanoparticles adhering to tumor vessels release cytotoxic drugs that internalize to the tumor tissue and cause cell death when the drug concentration reaches a therapeutic value. In the previous section, optimal nanoparticle sizes and avidities were obtained that maximize nanoparticle accumulation in tumors and minimize tumor size, considering fixed properties of the drugs carried by the nanoparticles. The spatial distribution of the drug molecules, however, depends on how far they transport in the tissue. We now consider the drug diffusivity as a design variable to be optimized, allowing determination of the optimal spatial drug distribution in the tissue, and thus with the potential to induce further cell death. Since the drug concentration profile in the tissue depends on the location of nanoparticles in the vessels (source of release), the drug diffusion coefficient *D* is optimized simultaneously with the nanoparticle size and avidity with the goal to achieve further tumor regression. This enables integrating the optimal selection of a drug property and nanoparticle design. The following optimization problem is formulated as an extension to that in Eq. (1).

3 $$\begin{array}{cc}\mathop{min}\limits_{d,\,\alpha ,D}\, & TD\\  & 1\le d\le 1000\,(nm)\\ s.t. & {10}^{10}\le \alpha \le {10}^{12}\,({m}^{-2})\\  & {10}^{-6}\le D\le {10}^{-3}\,(m{m}^{2}/s)\end{array}$$

Note that only the TD minimization problem is considered for the selection of drug diffusivity, since this design variable does not affect nanoparticle accumulation but only the transport of drug in the tissue after the nanoparticles adhere to the vasculature. A wide range of diffusion coefficients are considered in the optimization study accounting for small (*D*~10^−3^ mm^2^/s as upper bound, similar to oxygen) and large (*D*~10^−6^ mm^2^/s) molecules. As the diffusivity increases, the drug distributes deeper into the tissue. One may anticipate that if nanoparticle diameter and binding affinity provide uniform nanoparticle distribution in the tissue, and if a tumor is well vascularized, then a small value of diffusivity is desired (drug stays close to release site). On the other hand, if the nanoparticle design variables (size and avidity) do not provide sufficient uniformity in distribution of nanoparticles in the tumor vessels, the optimal diffusion coefficient of the drug should be high to allow it to reach cells farther from the vessels.

**Figure 2:**
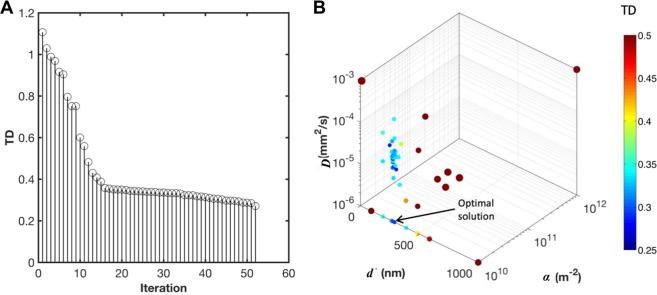
Optimization of nanoparticle size, avidity, and drug diffusivity. (**A**) Progress of MADS in obtaining the optimal solution. (**B**) Trial points selected by MADS, where the color and size of the points correspond to the value of TD. The smallest point refers to the optimal solution of TD minimization problem (shown by the arrow).

The optimization problem in Eq. (3) is solved using mesh adaptive direct search (MADS): only 52 blackbox evaluations (out of millions of possible combinations of design variables) were needed to converge to the minimal value of TD (Fig. 2(A)). The trial points selected by MADS are shown in Fig. 2(B). The optimal value of the variables is d* = 229 nm, α* = 10e10 m^−2^ and D* = 10^−6^ mm^2^/s. The associated value of TD is 26% representing a 2-fold increase in tumor regression as compared to the case where the drug diffusivity was not included in the design variables.

### Multi-objective optimization of tumor diameter and nanoparticle accumulation with respect to nanoparticle size

In certain ranges of nanoparticle diameters, tumor regression and nanoparticle accumulation at the tumor are two competing objective functions: optimizing one of them may compromise the other. As shown for the tumor and nanoparticle conditions in^27^, a nanoparticle of diameter of 288 maximizes the nanoparticle accumulation in the tumor, but a larger nanoparticle (334 nm) is needed to minimize the tumor size. The range of nanoparticle diameters between 288 nm and 334 nm represents a tradeoff between tumoral nanoparticle accumulation and tumor regression. Multiple optimal nanoparticle designs that quantify this tradeoff between TD and TNP were generated by solving the multiobjective optimization problem

4 $$\begin{array}{cc}\mathop{min}\limits_{d,\,\alpha }\, & [TD,-TNP]\\ s.t. & 1\le d\le 1000\,(nm)\end{array}$$

**Figure 3:**
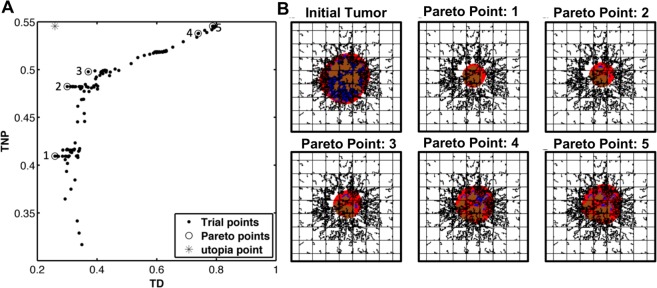
Solution of the multiobjective optimization problem. (**A**) Trial points selected by MADS to generate the Pareto front. (**B**) Display of the tumor at treatment initiation and after treatment using the optimal designs identified (quantified in Table 2), respectively showing a decrease of 74%, 70%, 63%, 26% and 21% in tumor diameter ratio (TD). The corresponding nanoparticle accumulation in the treated tumors (TNP) is 41%, 48%, 50%, 54% and 55%, respectively. Colors are as in Fig. 1.

The solution of the problem in Eq. (4) is a set of nanoparticle diameters. The utopia point is the unattainable design that optimizes both TNP and TD simultaneously. It lies on the upper left corner of the plot in Fig. 3(A), which also shows all the trial points selected by MADS during the convergence to the optimal designs. We identify 5 optimal diameters at the Pareto front. Table 2 lists the values of these diameters as well as the corresponding value of each objective function, TD and TNP. Note that as the nanoparticle diameter increases, TNP favorably increases and TD unfavorably increases. Similarly, as the nanoparticle diameter decreases, TD is enhanced at the cost of deceasing TNP. However, since the optimal designs are selected from the Pareto front, the compromise of the two objective functions is minimal. Figure 3(B) qualitatively displays the tumor regression corresponding to these values.

**Table 2:** Optimal nanoparticle sizes obtained by solving the multiobjective optimization problem in Eq. (4) showing the resulting values of TD and TNP. TD: ratio of tumor diameter after treatment to diameter at start of treatment; TNP: percent of injected nanoparticles that accumulate in tumor tissue.

Optimizer	d* (nm)	TD (%)	TNP (%)
1	229	26	41
2	451	30	48
3	532	34	50
4	900	74	54
5	1000	79	55

## Discussion

We extend our previous work on numerical optimization of nanoparticles for cancer nanotherapy to generate Pareto-optimal, tumor size-specific nanoparticle designs. Previously, we utilized optimization to find nanoparticle sizes and avidities that minimize tumor size and maximize tumoral nanoparticle accumulation^27^, enhancing both factors as compared with previous results without the use of optimization^25^. Here, we investigate the effect of tumor size on the optimal nanoparticle design, as size is an important indicator of clinical staging of the disease. A virtual cohort of tumors is grown to different sizes, and for each tumor two separate optimization problems are solved: one to minimize tumor diameter and the other to maximize tumor nanoparticle accumulation. Results showed that minimization of different tumor sizes was associated with different nanoparticle designs. Using a 2^nd^-order polynomial fit, a monotonically decreasing relation between tumor size and optimal nanoparticle diameter to be administered was identified (Fig. 1(B,C)). This result is consistent with previous studies^35–37^ that support the benefit of increased nanoparticle circulation time in larger tumors.

This optimization study presents a quantitative methodology to support clinical decisions based on tumor specific properties. The optimization problem that minimizes tumor size informs optimal selection of therapeutic nanoparticles that carry anticancer agents to tumors. The second optimization problem maximizes tumoral nanoparticle accumulation. The obtained designs may be suitable for enhancing tumor detection in non-invasive imaging^38^ or florescence-guided surgery^39^. In addition, the maximizers of tumoral accumulation could be used as second-line treatment to control tumor size after initial regression should the drugs become a toxicological risk, as discussed in^27^, and potentially as neoadjuvant treatment before resection.

We further investigated the possibility of increasing tumor regression by optimizing drug diffusivity along with nanoparticle size and avidity. How long nanoparticles circulate in blood vessels and at which location of the vasculature they adhere depends on multiple factors, such as hemodynamic forces, drift of nanoparticles from blood streamlines toward the endothelial layer, and nanoparticle-endothelial contact area^5^. These factors could be manipulated by changing nanoparticle physiochemical properties, primarily size and surface ligand density. This provides a potential pathway to optimize nanoparticle distribution in blood vessels, i.e., achieving high percentage of tumoral accumulation with adequate spatial uniformity within tumor vasculature. Subsequently, after drug is released from the vasculature-bound nanoparticles and internalizes into tissue, it has to overcome typically complex stromal conditions and cellular barriers to internalize in tumor cells. Here, drug properties such as diffusivity become predominant factors, as in pharmacokinetic/pharmacodynamic models^40^. Therefore, in nanoparticle-mediated drug delivery, drug molecules are subject to two sequential modes of transport: the nanoparticle transport stage, and tissue diffusion stage. Although these two stages are strongly dependent since drug profiles within tissue depend on nanoparticle location in blood vessels (source of drug release), to our knowledge, these two problems have been traditionally tackled separately, with one type of models focusing on nanoparticle design and the other on drug kinetics.

The proposed method enables simultaneous optimization of nanoparticle design and drug properties, with the results indicating that optimal selection of nanoparticles is drug dependent. In particular, we solved a 3-variable single objective program for finding nanoparticle size, ligand avidity, and drug diffusivity that minimizes tumor size. The optimal values of nanoparticle avidity and drug diffusivity were low, yet the nanoparticle size was moderate. The results showed that a nanoparticle with d* = 229 nm, α* = 10e^10^m^−2^, and D* = 10e^−6^ mm^2^/s decreases the simulated tumor diameter to 25% of its value before treatment. This represents a 2-fold increase in tumor regression when compared to the previous value when the drug diffusivity was not considered in the nanoparticle design. In comparison with the previous design, the new optimal size a slightly lower (229 versus 334 nm), while the nanoparticle avidity and drug diffusivity are at their lower bound. This suggests that better treatment efficacy may be reached with (i) relatively small nanoparticles (extended circulation time) that are large enough to carry adequate drugs, (ii) low nanoparticle avidity that seems to be most relevant with the obtained nanoparticle size in achieving optimal distribution in tumor blood vessels, and (iii) slow diffusing drug molecules that mostly reach cells close to blood vessels.

Although larger particles have stronger accumulation propensity, they tend to bind fairly quickly after they enter the tumor vasculature, in agreement with^41–43^, which reported that larger nanoparticles have faster margination toward blood vessels and stronger binding affinity. This situation may be desirable when tumors are small and poorly vascularized, as the particles would act as sources of drug surrounding the lesions. As tumors increase in size, nanoparticles need to stay in circulation longer in order to reach the tumor core. In this case, smaller nanoparticles may be advantageous. Previous work^26^ provides evidence that large particles have high accumulation rates but heterogeneous spatial distributions, since they tend to attach in the tumor peripheral tissue. In contrast, small nanoparticles have low binding affinities, but may distribute more uniformly within tumor tissue.

In addition, the obtained optimal value of *D* is low, providing a theoretical benefit by setting up targets for the selection or design of drugs. It is worth noting that this value of *D* could be influenced by tumor vascular density, as less vascularized tumors may require larger drug diffusivity to allow for deeper tissue penetration. Another parameter that may affect the optimal drug diffusion coefficient is the cellular uptake rate. Drugs with a strong binding affinity need to diffuse farther in the tissue to overcome cellular barriers and reach non-vascularized regions. In this case, the optimal value of the drug diffusion coefficient is expected to be larger. While controlling drug properties may be technically more difficult than the synthesis of particular nanoparticles, it may be possible to achieve desired drug diffusivities by considering the structure-activity relationship during the drug discovery process^44^. The optimization problem formulation in Eq. (3) provides a platform for integrating nanoparticle and drug properties during drug development. For instance, the results suggest that if drug diffusivity is low, normalizing tumor vessels before the administration of nanoparticles with cytotoxic agents may enhance cell death^45^. Future extension of this study may include augmenting more design variables such as nanoparticle shape, drug potency, and drug half-life, and studying how optimal values of these design variables vary using a heterogeneous cohort of tumors.

The tradeoff between tumoral nanoparticle accumulation and tumor regression was quantified. Nanoparticle diameter was treated as a design variable while fixing nanoparticle avidity and drug diffusivity to optimal values obtained earlier, leading to substantial decrease in the computational cost. Solving the bi-objective optimization problem, five plausible nanoparticle designs were identified at the Pareto front; in particular, nanoparticles with diameters [229, 451, 532, 691, 900, 1000] nm. Each of these sizes is associated with different values of TD and TNP. Since the solution belongs to the Pareto front, enhancement in one objective function causes minimal compromise to the other. The maximal accumulation of nanoparticles is 55% and can be reached with d* = 1000 nm, at which the tumor diameter decreases to 79% of its initial value. On the other end of the Pareto front is the minimizer of tumor diameter, which is a nanoparticle diameter of 229 nm causing the tumor to decrease to 25% of its initial value, and when used, 41% of nanoparticles adhere to the tumor.

Examining the limits of the Pareto front, we conclude that the decrease in tumor diameter is more sensitive to nanoparticle size than to nanoparticle accumulation. Using 229 nm nanoparticles instead of 1000 nm, a 3-fold increase in tumor regression was gained while nanoparticle accumulation was reduced by only 14%. A more detailed insight can be obtained by evaluating the shape of the Pareto front, which is of a concave nature. The smaller the nanoparticle, the more sensitive TD is to nanoparticle diameter, i.e., in this region, the enhancement in TD is considerably larger than the decrease in TNP. In contrast, a small compromise in TD causes large increases in TNP when the nanoparticles are large. Therefore, when tumor size decrease is the main criterion in the nanoparticle design, the minimizer of TD is d* = 229 nm when the tumor diameter is 760 μm. However, when drugs with low lethal doses are used, high tumor targeting is required. In this case, the results suggest the use of large nanoparticles (d* = 1000 nm). These extreme scenarios often exist when the objective of the study is well defined. In this case, the optimal nanoparticle diameters can be determined at a significantly lower computational cost using single objective optimization problems. On the other hand, when a balance between treatment toxicity and efficacy is required, the use of a nanoparticle size that lies at a non-steep region of the Pareto front may be appropriate. In this study, this region is close to the utopia point. For instance, the use of 451 nm nanoparticles yielded 70% tumor regression and 32% tumoral nanoparticle accumulation, representing a 2-fold enhancement in both objective functions as compared to the results previously obtained in^27^, for which drug diffusivity was not optimized.

The methodology presented herein offers the possibility to generate a library of nanoparticle specifications for personalized treatment, with each nanoparticle potentially serving a different purpose. Nanoparticles that have stronger weight on minimizing tumor volume could be suitable for first cycle treatment. Since these nanoparticles may exert higher toxicity, there is the option of using larger nanoparticles that maintain the treatment with lower toxicity. A library of nanoparticles obtained via the multiobjective optimization problem could help to adapt the therapy based on the tumor response to the earlier treatment cycles. Further, a multicriteria treatment design could provide a useful approach to the broad field of mathematical oncology. Studies have been performed to find protocols considering other factors that include in addition to tumor burden and tumor targeting, drug resistance^46^, total drug dose (toxicity)^47^, and immune response^48^. Using multiobjective optimization, two or more criteria could be incorporated in treatment planning or drug discovery, enabling the tackling of prognostic and toxicologic challenges.

The framework presented here is multiparametric, and the optimization results have different sensitivities to parameter values based on the parameter type and mechanisms involved. The optimal design of vasculature-adhering nanoparticles is expected to be affected mostly by tumor vasculature parameters such as the rate of production of new blood vessels, vessel size, and flow properties. These parameters directly contribute to the margination and binding of the particles. Other parameters that do not pertain to the vasculature are expected to have low or no effect on the optimal values of nanoparticle size and avidity. On the other hand, the optimal value of the drug diffusion coefficient is expected to be sensitive to the tissue parameters such as ECM density and mitosis rate. It is worth noting that the presence of sensitive parameters does not weaken the optimization results; in contrast, it enforces the use of the optimization framework to obtain optimal designs quickly based on individual tumor characteristics. In general, model parameters can be lumped into a clinically or biologically measurable variable such as the initial tumor size, proliferation rate, and hypoxia profiles, forming a set of potential biomarkers that can be used to stratify tumors and design optimal personalized therapies. In this study, we have demonstrated the use of the initial tumor size as a covariate for nanoparticle diameter selection. Future work may evaluate the predictive power of each tumor parameter using statistical analyses that include quantifying the predictor importance estimates^49^ and ranking the parameters using methods such as k-nearest neighbor or minimum redundancy maximum relevance (MRMR) algorithms.

New methods that apply machine learning to develop personalized treatment using population-wide data are classically based on protocols that may be successful but not necessarily optimal^50^. The methodology presented here could be used to develop a database of optimal treatment protocols, incorporating other clinical information – besides tumor size – with possible optimization of treatment parameters such as drug potency, ligand-receptor binding affinity, and drug kinetics. Longer term, a system could be generated to identify ideal treatment parameters. In addition, the design of optimal nanoparticles could be adapted as the tumor progresses. The results would be subject to preclinical validation, and an optimal database could be progressively updated when a new tumor is treated by an optimal therapy, providing a more promising predictor matrix for machine learning-based treatment planning.

## Conclusion

A preclinical computational study based on numerical optimization is presented to establish a methodology to determine optimal nanotherapy parameters. Building upon our previous work^26,27^, this study investigates the effect of initial tumor size on the optimal selection of nanoparticles, and the effect of drug diffusion on nanoparticle design optimization, and quantifies the tradeoff between nanotherapy therapeutic and toxicological metrics. The results offer the potential to improve efficacy of nanoparticle-mediated anticancer drug delivery, and reveal nanoparticle sizes with the potential to efficiently treat a range of tumor sizes. A possible extension of this work includes generating a library of nanoparticles optimized for different tumor model parameters, correlating the biological parameters to key clinicopathological variables, and developing machine-learning models to identify optimal nanoparticle designs customized to patient tumor-specific parameters. In addition, the nanoparticle drug delivery considers a 2D tumor, neglects the immune response to therapy, and focuses on spherical nanoparticles. Extending the computational model would allow further optimization studies related, for example, to combining immune checkpoint inhibitors with cytotoxic drugs, optimizing nanoparticle physiochemical properties, and coupling with patient tumor-specific omics data.

## Materials and Methods

**Figure 4:**
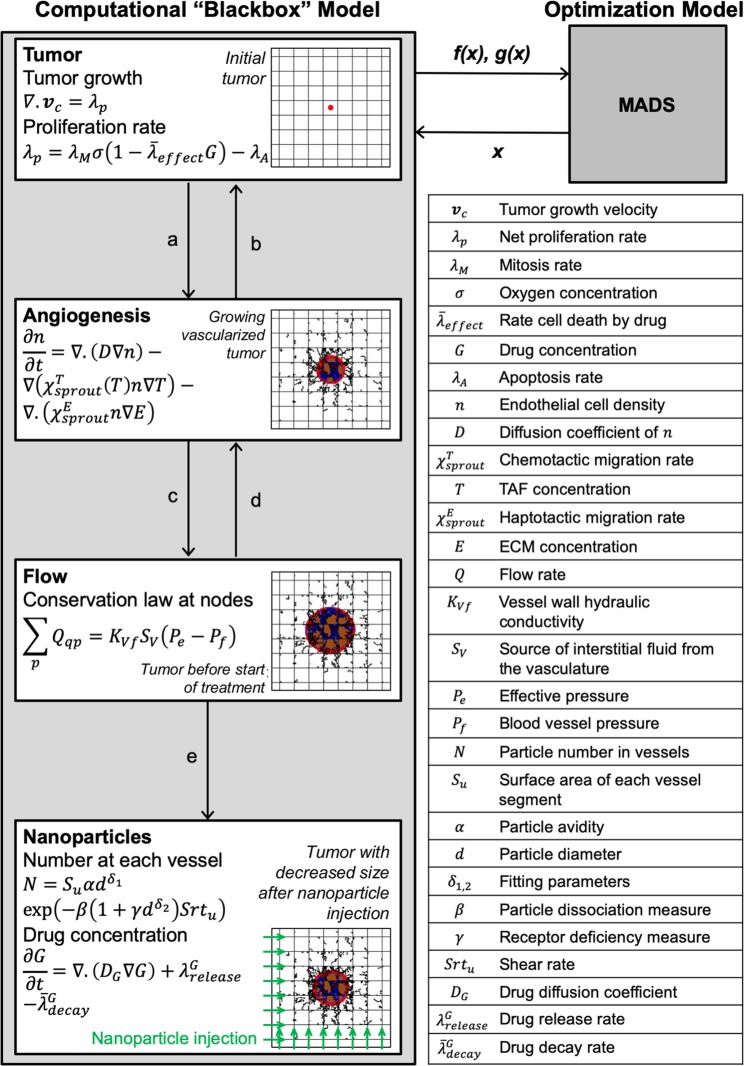
Hybrid framework for optimizing nanoparticle mediated drug delivery. The framework couples a computational “blackbox” model of vascularized tumor growth and nanoparticle treatment^7,25^ with an optimization model based on the mesh adaptive direct search (MADS) algorithm^26,27^. The computational model^25^ uses a nanoparticle design (*x*) selected by MADS and evaluates the objective function *f(x)* and the constraints *g(x)*. These values are used by MADS to recommend a new trial point for the computational model. This process iterates until the objective function is optimized, so that the result of the hybrid framework generates optimal nanoparticle designs x^*^. In the computational model, multiple sub-models interact and exchange parameters, including: (**a**) angiogenic factors; (**b**) oxygen and nutrients; (**c**) location of capillary junctions; (**d**) wall shear stress, flow stimulus, and intravascular pressure; and (**e**) vessel radii, vessel surface areas, and flow rate. The tumor is displayed in the computational model at inception, after vascularization, before treatment and after treatment. Colors are as in Fig. 1.

The computational model of nanoparticle and drug transport in tumor tissue described in^25^ is used here as a “blackbox” system. The model evaluates tumor regression and tumoral nanoparticle accumulation as a function of nanoparticle parameters, which include nanoparticle size and binding affinity. The model is used by an optimization algorithm to systematically evaluate different combinations of input variables to determine which ones optimize the outputs of interest. In general, the interaction between the two models is as following: starting from an initial guess of the nanoparticle design, the computational model evaluates the objective (goal) function and the (restricting) constraints. This quantitative information is used by rigorous algorithms to select new trial points from the design space with the goal of outperforming the current (incumbent) iteration. This process is repeated until an algorithmic termination criterion is met. The termination criterion could be to attain a small change in the objective function or in the values of the design variables. The optimization framework is depicted schematically in Fig. 4.

### Computational model

The main equations of the computational “blackbox” model are shown in Fig. 4. The initial condition of the computational model in^25^ is a 2 × 2 mm vascularized through which blood enters from the bottom and left sides. An avascular cancerous lesion of initial diameter 100 um is placed at the center of the domain. Oxygen and nutrients are simulated to be delivered from the nearby blood vessels. A set of PDEs are used to model proliferation of the viable region and formation of hypoxia and necrosis. A grid-based discrete model of angiogenesis^12^ is used to determine the formation of the new blood vessels that sprout from existing vessels in response to hypoxia in tumor tissue^51,52^. In the case of small micrometastases, it has been shown that the surrounding vasculature begins to rework itself in response to the hypoxia within these metastases^53^. Accordingly, in the model, the development of blood vessels is driven by a hypoxic landscape that produces tumor angiogenic factors and promotes the development of neovasculature sourcing from existing vessels. If the new vessels penetrate into the tumor, they enable the lesion to grow larger by becoming vascularized.

Nanoparticles are injected downstream through the vasculature once the tumor reaches a diameter (760 μm) sufficient for evaluation of therapeutic effects^7^. The nanoparticles adhere to the tumor vessel walls through ligand-receptor pairs. The success of binding is challenged by blood dissociative forces. The percent accumulation and the spatial distribution of nanoparticles depends on the nanoparticle physiochemical properties, mainly size and binding affinity^7^. Once a nanoparticle adheres to the vessel wall, the drug is released to the tissue at a rate that is proportional to the nanoparticle size^25^. The drug transport is modeled by a reaction-diffusion equation, causing tumor regression (see Fig. 4). As a representative cell-cycle dependent drug is simulated, the rate of cell death in the proliferation rate equation is assumed proportional to the drug potency, represented by the parameter ${\bar{\lambda }}_{effect}$. This parameter is linked to the half maximal inhibitory concentration (IC_50_) by determining which value causes an avascular simulated tumor to shrink by half its pre-treatment size within a certain timeframe (as was done in^14^). Thus, drugs of lower potency have higher IC_50_. Following our previous work in^25^, this study considers a drug with potency similar to that of Paclitaxel. Our previous simulation of the model in^27^ indicated that a simulation of 1 day is adequate to evaluate the performance of different nanoparticle designs. Therefore, the simulation is stopped 1 day after injection and record the tumor size and percent of nanoparticle accumulation in the tumor tissue.

### Optimization model

We develop an optimization model that systematically simulates the computational model. Here, the computational model inputs are classified into variables (*x*) to be optimized and parameters (*p*) to be held at fixed values through the optimization process. A key variable considered in this study is the nanoparticle diameter. The binding affinity of nanoparticles and the diffusivity of the cytotoxic drug are additionally considered. The set of parameters include other model characteristics such as drug potency, blood vessel properties, and tumor proliferation rate. The main parameters are listed in Table 3.

**Table 3:** Main parameters used in the computational “blackbox” model.

Model parameter	Value
Drug decay rate^27^	4.1588 s^−1^
Drug diffusivity^27^	3.334 × 10^–6^ mm^2^/s (*)
Nanoparticle avidity^25^	2.95 × 10^10^ m^−2^
Nanoparticle dissociation tendency^25^	6.63 × 10^–4^ m^−2^.s
Measure of receptor deficiency^25^	1.07 × 10^3^ m^−1.57^

(*) The value for drug diffusivity was incorrectly reported in Table 1 of^27^ as 3.334 × 10^−3^ mm^2^/s, and is corrected here. Both the study in^27^ and here used the same default value.

The model produces multiple outputs that relate to nanoparticle design performance in terms of antitumor activity and targeted regions. Two objective functions are defined: the ratio of tumor diameter at the end of the treatment normalized by the diameter at the start of treatment (TD), and the percentage of injected nanoparticles that adhere to tumor vessels (TNP). The objective of the optimization model is to minimize TD and maximize TNP. In addition, we are interested in computing the tradeoff between TD and TNP, as these are competing objective functions^27^. A multiobjective optimization formulation is used to generate a set of optimal nanoparticle designs that define the Pareto front, i.e., the set of points for which one objective cannot be improved without deteriorating the other.

All optimization problems are solved using the Mesh Adaptive Direct Search (MADS) algorithm^54^. MADS is a derivative-free optimization algorithm that does not require gradient evaluation. This class of algorithms is suitable when using computational models as “blackboxes” for which the gradients are either unavailable or require unjustifiable amount of effort and time to be approximated. Moreover, the model is computationally expensive due to strong coupling between the submodels; each “blackbox” evaluation requires 1.5 of hours of CPU time on an Intel(R) Core(TM) i7–3770 CPU @ 3.4 GHz processor. To address this complexity, a surrogate-assisted optimization approach is used to learn from previous trials while iterating towards the optimal solution. In this approach, a surrogate of the model is created and continuously updated to facilitate the search step of MADS [23]. The surrogate approximation is combined with evaluations of the “blackbox” model at the poll step of MADS, providing a fast and rigorous convergence to the optimal nanoparticle design. MADS is implemented in the NOMAD C++ software package^55^. The MATLAB interface is used to communicate with NOMAD and automatically exercise the “blackbox” tumor model.

Since the analysis model is a “blackbox,” we cannot know whether the underlying optimization problem functions are convex. It is thus impossible to prove formally that the obtained optimal solutions are global. However, we previously examined the behavior of the objective functions with respect to the nanoparticle diameter, and both of them exhibited an empirical convex profile (see Fig. 2(a,b) in^27^). Since the other variables were boundary optimizers, the uniqueness of the optimal solution within the defined design space is supported with extremely high likelihood.

## Data Availability

All data analyzed during this study are included in this published article.

## References

[1] American Cancer Society. *Cancer Facts & Figures*, https://www.cancer.org/content/dam/cancer-org/research/cancer-facts-and-statistics/annual-cancer-facts-and-figures/2019/cancer-facts-and-figures-2019.pdf (2019). 10.6004/jadpro.2020.11.2.1 PMC7848816 33532112

[2] Gabizon A, Shmeeda H, Barenholz Y (2003). Pharmacokinetics of pegylated liposomal Doxorubicin: review of animal and human studies. Clin Pharmacokinet.

[3] Hare JI (2017). Challenges and strategies in anti-cancer nanomedicine development: An industry perspective. Adv Drug Deliv Rev.

[4] Sen Gupta A (2016). Role of particle size, shape, and stiffness in design of intravascular drug delivery systems: insights from computations, experiments, and nature. Wiley Interdiscip Rev Nanomed Nanobiotechnol.

[5] Decuzzi P, Ferrari M (2006). The adhesive strength of non-spherical particles mediated by specific interactions. Biomaterials.

[6] Decuzzi P, Lee S, Bhushan B, Ferrari M (2005). A theoretical model for the margination of particles within blood vessels. Ann Biomed Eng.

[7] Frieboes HB, Wu M, Lowengrub J, Decuzzi P, Cristini V (2013). A computational model for predicting nanoparticle accumulation in tumor vasculature. PLoS One.

[8] Kumar A, Graham MD (2011). Segregation by membrane rigidity in flowing binary suspensions of elastic capsules. Phys Rev E Stat Nonlin Soft Matter Phys.

[9] Fronczyk K, Kottas A (2014). A Bayesian approach to the analysis of quantal bioassay studies using nonparametric mixture models. Biometrics.

[10] Lee TR (2014). Quantifying uncertainties in the microvascular transport of nanoparticles. Biomech Model Mechanobiol.

[11] van de Ven AL (2012). Integrated intravital microscopy and mathematical modeling to optimize nanotherapeutics delivery to tumors. AIP Adv.

[12] Wu M (2013). The effect of interstitial pressure on tumor growth: coupling with the blood and lymphatic vascular systems. J Theor Biol.

[13] Wu M (2014). The effect of interstitial pressure on therapeutic agent transport: coupling with the tumor blood and lymphatic vascular systems. J Theor Biol.

[14] Curtis LT, England CG, Wu M, Lowengrub J, Frieboes HB (2016). An interdisciplinary computational/experimental approach to evaluate drug-loaded gold nanoparticle tumor cytotoxicity. Nanomedicine (Lond).

[15] Curtis LT, Rychahou P, Bae Y, Frieboes HB (2016). A Computational/Experimental Assessment of Antitumor Activity of Polymer Nanoassemblies for pH-Controlled Drug Delivery to Primary and Metastatic Tumors. Pharm Res.

[16] Leonard F (2017). Macrophage Polarization Contributes to the Anti-Tumoral Efficacy of Mesoporous Nanovectors Loaded with Albumin-Bound Paclitaxel. Front Immunol.

[17] Leonard F (2016). Enhanced performance of macrophage-encapsulated nanoparticle albumin-bound-paclitaxel in hypo-perfused cancer lesions. Nanoscale.

[18] Leonard, F. *et al*. Nonlinear response to cancer nanotherapy due to macrophage interactions revealed by mathematical modeling and evaluated in a murine model via CRISPR-modulated macrophage polarization *Cancer Immunol Immunoterapy* **69**, 731–744, 10.1007/s00262-020-02504-z (2020). 10.1007/s00262-020-02504-z PMC7186159 32036448

[19] Miller HA, Frieboes HB (2019). Evaluation of Drug-Loaded Gold Nanoparticle Cytotoxicity as a Function of Tumor Vasculature-Induced Tissue Heterogeneity. Ann Biomed Eng.

[20] Miller HA, Frieboes HB (2019). Pharmacokinetic/pharmacodynamics modeling of drug-loaded PLGA nanoparticles targeting heterogeneously vascularized tumor tissue. Pharmaceutical Research.

[21] England CG, Ng CF, van Berkel V, Frieboes HB (2015). A Review of Pharmacological Treatment Options for Lung Cancer: Emphasis on Novel Nanotherapeutics and Associated Toxicity. Curr Drug Targets.

[22] Curtis LT, Frieboes HB (2016). The Tumor Microenvironment as a Barrier to Cancer Nanotherapy. Adv Exp Med Biol.

[23] Sharma, N., Sharma, M., Sajid Jamal, Q. M., Kamal, M. A. & Akhtar, S. Nanoinformatics and biomolecular nanomodeling: a novel move en route for effective cancer treatment. *Environ Sci Pollut Res Int*, 10.1007/s11356-019-05152-8 (2019). 10.1007/s11356-019-05152-8 31025282

[24] Chamseddine, I. M. & Rejniak, K. A. Hybrid modeling frameworks of tumor development and treatment. *Wiley Interdiscip Rev Syst Biol Med* **12**, e1461, 10.1002/wsbm.1461 (2020). 10.1002/wsbm.1461 PMC6898741 31313504

[25] Curtis LT, Wu M, Lowengrub J, Decuzzi P, Frieboes HB (2015). Computational Modeling of Tumor Response to Drug Release from Vasculature-Bound Nanoparticles. PLoS One.

[26] Chamseddine, I. M. & Kokkolaras, M. Nanoparticle Optimization for Enhanced Targeted Anticancer Drug Delivery. *J Biomech Eng* **140**, 10.1115/1.4038202 (2018). 10.1115/1.4038202 29049542

[27] Chamseddine IM, Frieboes HB, Kokkolaras M (2018). Design Optimization of Tumor Vasculature-Bound Nanoparticles. Sci Rep.

[28] Ludwig JA, Weinstein JN (2005). Biomarkers in cancer staging, prognosis and treatment selection. Nat Rev Cancer.

[29] Forster JC, Harriss-Phillips WM, Douglass MJ, Bezak E (2017). A review of the development of tumor vasculature and its effects on the tumor microenvironment. Hypoxia (Auckl).

[30] Halaoui R (2017). Progressive polarity loss and luminal collapse disrupt tissue organization in carcinoma. Genes Dev.

[31] Kai F, Drain AP, Weaver VM (2019). The Extracellular Matrix Modulates the Metastatic Journey. Dev Cell.

[32] Lynch CM (2017). Prediction of lung cancer patient survival via supervised machine learning classification techniques. Int J Med Inform.

[33] Lynch CM, van Berkel VH, Frieboes HB (2017). Application of unsupervised analysis techniques to lung cancer patient data. PLoS One.

[34] Mahlbacher GE, Reihmer KC, Frieboes HB (2019). Mathematical modeling of tumor-immune cell interactions. J Theor Biol.

[35] Schmid G, Kreyling WG, Simon U (2017). Toxic effects and biodistribution of ultrasmall gold nanoparticles. Arch Toxicol.

[36] Guo D (2017). Riboflavin-containing telodendrimer nanocarriers for efficient doxorubicin delivery: High loading capacity, increased stability, and improved anticancer efficacy. Biomaterials.

[37] Lima AC, Alvarez-Lorenzo C, Mano JF (2016). Design Advances in Particulate Systems for Biomedical Applications. Adv Healthc Mater.

[38] Kinsella JM (2011). X-ray computed tomography imaging of breast cancer by using targeted peptide-labeled bismuth sulfide nanoparticles. Angew Chem Int Ed Engl.

[39] Hill TK, Mohs AM (2016). Image-guided tumor surgery: will there be a role for fluorescent nanoparticles?. Wiley Interdiscip Rev Nanomed Nanobiotechnol.

[40] Danhof M, de Lange EC, Della Pasqua OE, Ploeger BA, Voskuyl RA (2008). Mechanism-based pharmacokinetic-pharmacodynamic (PK-PD) modeling in translational drug research. Trends Pharmacol Sci.

[41] Muller K, Fedosov DA, Gompper G (2014). Margination of micro- and nano-particles in blood flow and its effect on drug delivery. Sci Rep.

[42] Lee TR (2013). On the near-wall accumulation of injectable particles in the microcirculation: smaller is not better. Sci Rep.

[43] Cooley M (2018). Influence of particle size and shape on their margination and wall-adhesion: implications in drug delivery vehicle design across nano-to-micro scale. Nanoscale.

[44] Guha R (2013). On exploring structure-activity relationships. Methods Mol Biol.

[45] Chauhan VP (2012). Normalization of tumour blood vessels improves the delivery of nanomedicines in a size-dependent manner. Nat Nanotechnol.

[46] Boldrini JL, Costa MI (2000). Therapy burden, drug resistance, and optimal treatment regimen for cancer chemotherapy. IMA J Math Appl Med Biol.

[47] Glick, A. E. & Mastroberardino, A. An optimal control approach for the treatment of solid tumors with angiogenesis inhibitors. *Mathematics* **5** (2017).

[48] Cunningham JJ, Brown JS, Gatenby RA, Stankova K (2018). Optimal control to develop therapeutic strategies for metastatic castrate resistant prostate cancer. J Theor Biol.

[49] Breiman, L., Friedman, J., Olshen, R. & Stone, C. *Classification and Regression Trees*. (CRC Press, 1984).

[50] Rajkomar A, Dean J, Kohane I (2019). Machine Learning in Medicine. N Engl J Med.

[51] McDougall SR, Anderson AR, Chaplain MA (2006). Mathematical modelling of dynamic adaptive tumour-induced angiogenesis: clinical implications and therapeutic targeting strategies. J Theor Biol.

[52] Macklin P (2009). Multiscale modelling and nonlinear simulation of vascular tumour growth. J Math Biol.

[53] Li XF, O’Donoghue JA (2008). Hypoxia in microscopic tumors. Cancer Lett.

[54] Audet C, Dennis JJ (2006). Mesh adaptive direct search algorithms for constrained optimization. SIAM Journal on Optimization.

[55] Le Digabel S (2011). Algorithm 909: Nomad: Nonlinear optimization with the mads algorithm. ACM Transactions on Mathematical Software.

